# Sepsis: Diagnostic and Therapeutic Challenges

**DOI:** 10.1155/2016/5786182

**Published:** 2016-10-17

**Authors:** Zsolt Molnár, Evangelos J. Giamarellos-Bourboulis, Anand Kumar, Axel Nierhaus

**Affiliations:** ^1^Department of Anaesthesiology and Intensive Therapy, Faculty of Medicine, University of Szeged, 6 Semmelweis St., Szeged 6725, Hungary; ^2^4th Department of Internal Medicine, Attikon University Hospital, 1 Rimini Str., 12462 Athens, Greece; ^3^Sections of Critical Care Medicine and Infectious Diseases, Medical Microbiology and Pharmacology, University of Manitoba, Winnipeg, MB, Canada; ^4^Department of Intensive Care Medicine, University Medical Center Hamburg-Eppendorf, 20151 Hamburg, Germany

Intensive and critical care medicine has gone through unprecedented development over the last few decades. According to recent surveys, we now treat severalfold more critically ill patients in intensive care units (ICU) worldwide as compared to 10 years ago [1]. One of the most challenging tasks that intensive care specialists face is the treatment of serious infection-related multiple organ dysfunction, termed “sepsis” and “septic shock.” Sepsis has become a serious health economic issue around the world, with more patients dying due to sepsis related complications than breast and colorectal cancer together. According to recent data from the United States and Germany, sepsis is the single most expensive reason for hospitalization [2–4]. Large retrospective and prospective studies indicate that mortality of septic shock can still be as high as 45–55% and is associated with a 2- to 3-fold longer ICU and hospital stay [4, 5]. Accordingly, sepsis has become a serious health economic issue; hence, research of new frontiers in the diagnosis and treatment of sepsis has been a top priority in intensive care medicine.

The performance of sepsis research has had several difficulties. First of all, defining sepsis is a very difficult task as it is not a definitive disease [6]. Ever since the term “sepsis syndrome” was invented by Bone and coworkers, there has been a continuous search for appropriate, universally applicable definitions [7]. The latest consensus definitions have recently been published by an international task force as “Sepsis-3” [8]. Nonetheless, the problem lingers because sepsis is a very heterogeneous condition of different etiologies and severity which can range from a mild form of one organ system dysfunction requiring only moderate support to a very severe multiple system organ failure needing invasive salvage therapies. This heterogeneity of the investigated patient populations may, at least in part, explain why clinical research of the last 30 years has often been regarded as a failure, since most studies either failed to show clear survival benefit, or positive results of single center studies were later contradicted by large multicenter trials [9].

In addition to the problems of defining sepsis, serious challenges in diagnostics also exist. In contrast to other specialties where diagnostic laboratory and/or radiological tests with high sensitivity and specificity exist, the diagnosis of sepsis is more complicated. There are two main elements to this problem. On the one hand, organ dysfunction has to be recognized early and resuscitation measures must be commenced without delay in order to stabilize the patient and to avoid any secondary organ damage. Simultaneously, the nature of the underlying infection has to be clarified. Unfortunately, conventional indicators of infection (fever, leukocytosis, etc.) have poor performance in the critically ill. Even new biomarkers have only 75–85% sensitivity and specificity to diagnose infection at best, mainly because of the fact that pathobiology varies considerably from one patient to another [6]. Therefore, any single test is inadequate to make the diagnosis of sepsis, and it is highly unlikely that there will ever be a particular laboratory parameter that can do the job. Hence, the competence and responsibility of the attending physician are important beyond measure at present and may remain so for years to come.

Finally, it seems highly unlikely that a single comprehensive and specific “antisepsis” medication would appear on the scene. Treatment will always include nonspecific measures of organ support, such as oxygen therapy, mechanical ventilation, hemodynamic support, and renal replacement therapy, and antimicrobials. Of note, there seems to be a clear window of opportunity for most of these interventions to have an impact on survival: treatment has to be initiated as early as possible [10]. In addition, there is some rationale to apply adjunctive treatment and help the immune system in its deadly fight against the invading pathogens, by either reinforcing it or attenuating the inflammatory response [11]. However, this requires the introduction of novel markers of the immune response that enable the physician at the bedside to accurately gauge the actual state of the immune system and to tailor highly individualized interventions [12].

This issue tried to address some of these points. These new results and reviews, interesting as they are, may also serve as hypothesis generating for future research. In this special issue on sepsis, only a small bundle of the huge array of topics in novel sepsis research will be presented, but it nevertheless demonstrates the motivation and determination of the intensive care community in order to improve understanding and therapeutic modalities for our patients.

*Zsolt Molnár* *Evangelos J. Giamarellos-Bourboulis* *Anand Kumar* *Axel Nierhaus*
